# Roles and Mechanisms of Long Non-Coding RNAs in Breast Cancer

**DOI:** 10.3390/ijms24010089

**Published:** 2022-12-21

**Authors:** Jia Su, Lihao Deng, Yan-Dong Wang

**Affiliations:** State Key Laboratory of Chemical Resource Engineering, College of Life Science and Technology, Beijing University of Chemical Technology, Beijing 100029, China

**Keywords:** ncRNAs, lncRNAs, circRNAs, breast cancer

## Abstract

Breast cancer is a major health threat and the second leading cause of cancer-related deaths in women worldwide. The detailed mechanisms involved in the initiation and progression of breast cancer remain unclear. In recent years, amounting evidence indicated that long non-coding RNAs (lncRNAs) played crucial roles in regulating various biological processes and malignancy tumors, including breast cancer. In this review, we briefly introduce the functions and underlying mechanisms by which lncRNAs are involved in breast cancer. We summarize the roles of the lncRNAs in regulating malignant behaviors of breast cancer, such as cell proliferation, migration and invasion, epithelial–mesenchymal transition (EMT), apoptosis, and drug resistance. Additionally, we also briefly summarize the roles of circular RNAs (circRNAs) in breast cancer carcinogenesis.

## 1. Introduction

Breast cancer is one of the highly prevalent tumors worldwide. In the United States, breast cancer has the highest incidence among women and ranks the second in the leading cause of cancer-related deaths [1]. It is estimated that breast cancer may cause 281,550 new cases and 43,600 deaths in the USA in 2021 [1]. Due to improvements in the clinical treatment of breast cancer, including radiotherapy, chemotherapy, surgery, and endocrine treatments, the five-year relative survival rate for breast cancer can reach up to 80–92%, while the survival rate for metastatic breast cancer is still less than 25% [2,3]. Thus, it is urgent to figure out the detailed molecular mechanisms of breast cancer.

Long non-coding RNAs (lncRNA) represent a subtype of non-coding RNA (ncRNA) transcripts without protein-coding ability, which are longer than 200 nts in length [4]. Over the past few years, thousands of lncRNAs have been identified based on high-throughput RNA sequencing and bioinformatics analyses [5]. Moreover, numerous studies have revealed the crucial roles of lncRNAs in diverse cellular contexts and multiple biological processes, including cancers [6]. Based on the TCGA database, around 1059 dysregulated lncRNAs have been identified in breast cancer tissues [7]. More than that, plenty of studies have highlighted the particular functions of lncRNAs in the cell proliferation, apoptosis, cell metastasis and invasion, epithelial–mesenchymal transition (EMT), and drug resistance of breast cancer [8,9,10,11,12]. Moreover, lncRNAs have several clinical pathological features of breast cancer patients, such as tumor node metastasis (TNM) stage, tumor size, and lymph node metastasis [10,13,14]. Over the past few decades, studies on lncRNAs have revealed that the dysregulated lncRNAs in breast cancer are significantly associated with the survival rate and recurrence of breast cancer patients [11,15]. Thus, these characteristics have highlighted the potential use of lncRNAs for breast cancer diagnosis. These lncRNAs in breast cancer tissues may be developed as diagnostic biomarkers. Additionally, circular RNAs (circRNAs) represent a class of ncRNAs with a unique circular configuration in structure [16]. circRNAs can interact with RNAs and proteins to be involved in gene regulation at the transcription or post-transcription levels [17]. Furthermore, many studies have highlighted the critical roles displayed by circRNAs in breast cancer development.

**Figure 1:**
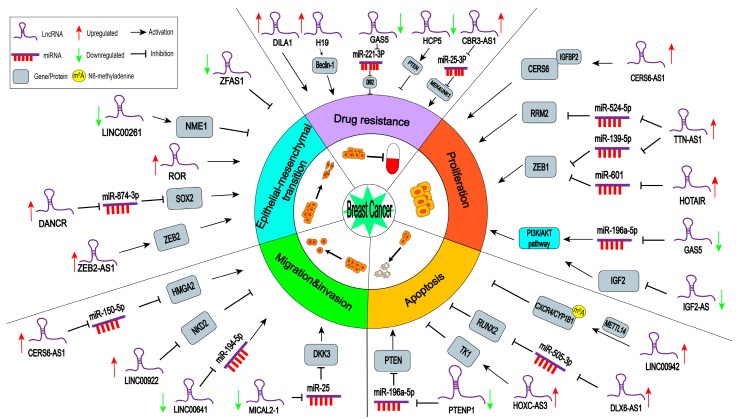
The roles and molecular mechanisms of lncRNAs in breast cancer. Selected lncRNAs have been found to be involved in breast cancer by affecting cell proliferation and apoptosis, cell migration and invasion, epithelial–mesenchymal transition (EMT), and drug resistance by manipulating those related target genes and signaling pathways. Abbreviations: *CERS6*, ceramide synthase 6; *CXCR4*, chemokine receptor 4; *CYP1B1*, cytochrome P450 1B1; *DKK2*, dickkopf 2; *DKK3*, dickkopf 3; *HMGA2*, high-mobility group protein 2; IGFBP2, insulin-like growth factor binding protein-2; IGF2, insulin like growth factor 2; m^6^A, N6-methyladenine; METTL14, methyltransferase-like 14; *NKD2*, naked cuticle homolog 2; *NME1*, NME/NM23 nucleoside diphosphate kinase 1; *PTEN*, phosphatase and tensin homolog; *RRM2*, ribonucleotide reductase subunit 2; *RUNX2*, runt-related transcription factor 2; *SOX2*, SRY-Box 2; *TK1*, thymidine kinase 1; *ZEB1*, zinc finger E-box binding homeobox 1; *ZEB2*, zinc finger E-box binding homeobox 2.

In this review, we will summarize the functions of lncRNAs in regulating breast cancer progression and highlight the molecular mechanisms by which lncRNAs are involved in breast cancer (Table 1 and Figure 1).

## 2. LncRNAs Are Correlated with Clinical Outcomes in Breast Cancer

Due to the high mortality and recurrence rate of breast cancer, precise treatment strategies for breast cancer patients are needed. Numerous studies show that some dysregulated lncRNAs are associated with clinical clinicopathological features of breast cancer [59]. Some lncRNAs can work as tumor suppressors in breast cancer. Gao et al. reported that phosphatase and tensin homolog pseudogene 1 (PTENP1) are expressed at low levels in breast cancer tissues, and its expression levels are negatively related to tumor stage [19] (Figure 1). Growth-arrest-specific transcript 5 (GAS5) levels are negatively associated with tumor size, histological grade, and lymph node metastasis [20].

By contrast, lncRNA Rac GTPase activating protein 1 pseudogene (RACGAP1P) is highly expressed in breast cancer tissues and cells. Higher RACGAP1P expression is associated with lymph node metastasis, distance metastasis, and TNM stage in breast cancer patients. Moreover, patients with higher RACGAP1P levels have a shorter survival time [22]. Highly upregulated in liver cancer (HULC), one of the dysregulated lncRNAs in multiple cancers, was observed to be overexpressed in breast cancer tissues, and associated with a poor overall survival rate [8]. Similarly, higher LncRNA distal-less homeobox 6 antisense 1 (DLX6-AS1) expression levels have been found in breast cancer tissues, and are also closely correlated with tumor size, lymph node metastasis, and clinical stage [23]. Bao et al. demonstrated that a higher expression of ceramide synthase 6 antisense RNA 1 (CERS6-AS1) is significantly associated with tumor grade, clinical stage, and poor prognosis in breast cancer patients [9]. According to Qiao and his colleagues, LINC00673 is upregulated in breast cancer tissues and cells. Moreover, its levels are closely associated with tumor size and a shorter overall survival time in breast cancer patients [13]. TROJAN has been found to be expressed at a high level in breast cancer tissues, and its expressing levels in breast cancer tissues are positively correlated with tumor size and pathologic grade [15]. Similarly, the increased expression of H19 and the MIR210 host gene (MIR210HG) is closely related to metastasis and tumor stage [11,24]. Lu et al. reported that high LINC00511 levels in breast cancer tissues and cells are significantly related to large tumors, TNM stages, and lymph node metastasis [14]. The increased expression of zinc finger E-box binding homeobox 2 antisense RNA 1 (ZEB2-AS1) has been observed in breast cancer tissues, and its levels are positively associated with lymph node metastasis and distant metastasis [25]. MIR200C and MIR141 host genes (MIR200CHG), endogenous bornavirus-like nucleoproteins (EBLN3P), and Linc00839 are closely related to a higher pathological grade, tumor size, and clinical stage of breast cancer patients [26,60,61]. Moreover, the high expressions of HOXC cluster antisense RNA 3 (HOXC-AS3), LINC02273, and maternally expressed gene 3 (MEG3) are strongly associated with TNM stage, lymph node metastasis, and metastasis [27,62,63]. In addition to a lack of reliable biomarkers, limited clinical treatment strategies also contribute to breast-cancer-related deaths. Thus, new biomarkers are urgently needed to enhance the diagnosis of breast cancer. The lncRNAs discussed above are closely related to the clinical outcomes of breast cancer patients, and may function as potential diagnostic biomarkers for breast cancer treatment in the future.

## 3. LncRNAs Regulate Cell Proliferation in Breast Cancer

The uncontrolled growth capability is one of the most significant characteristics of cancer cells. Amounting evidence has revealed that some lncRNAs have been involved in breast cancer cell proliferation. Interestingly, MIR210HG is highly expressed in breast cancer tissues, and its downregulation in MDA-MB-231 and MCF-7 inhibits cell proliferation by functioning as a miR-1226-3p ceRNA to derepress mucin1 (*MUC1-C*) [24]. Titin antisense1 (TTN-AS1), a highly expressed lncRNA in breast cancer tumors, has been found to facilitate cell proliferation in breast cancer by competitively sponging miR-524-5p to upregulate the ribonucleotide reductase subunit 2 (*RRM2*) [28]. Similarly, another study also reported that TTN-AS1 could promote the growth of breast cancer cells by targeting the miR-139-5p/zinc finger E-box binding homeobox 1 (*ZEB1*) axis [29]. In addition, the knockdown of H19 suppresses cell growth by attenuating miR-138-mediated SRY-box transcription factor 4 (*SOX4*) suppression in breast cancer [11]. These data indicate the key role of the H19/miR-138/*SOX4* axis in breast cancer. As reported by Qiao et al., LINC00673 silencing was found to repress the cell viability and tumor growth of breast cancer cells in vitro and in vivo. Mechanistic investigations showed that LINC00673 could sponge to miR-515-5p and consequently upregulate microtubule affinity-regulating kinase 4 (*MARK4*) expression, thus inactivating the Hippo signaling pathway [13].

Furthermore, a previous study demonstrated that HOTAIR was obviously overexpressed in breast cancer samples, and its inhibition repressed the cell growth both in vivo and in vitro [30]. Mechanistically, the oncogenic role of HOTAIR in breast cancer was mediated by the sponging of miR-601 and subsequently regulated *ZEB1* expression [30]. In addition, lncRNA SRY-Box 21 antisense RNA 1 (SOX21-AS1) was significantly upregulated in breast cancer specimens, and the silencing of SOX21-AS1 in MCF-7 and BT-20 cells retarded cell growth via PI3K/AKT pathways [31]. Gong et al. reported that knockdown of lung cancer progression-associated transcript 1 (LCPAT1) which suppressed cell proliferation and inhibited xenograft tumor formation in nude mice. Mechanistically, LCPAT1 could recruit retinoblastoma-binding protein 4 (RBBP4) to the promoter region of microfibril-associated protein 2 (*MFAP2*) to induce its expression, thus contributing to breast cancer [33]. Likely, lncRNA NEAT1 expression was upregulated in breast cancer tissues, and its inhibition in SKBR3 cells impaired cell proliferation by sponging miR-410-3p and subsequently downregulating cyclin D1 (*CCND1*) [34].

*MYC*, a famous oncogenic transcription factor, can bind to the promoter region of the DC-STAMP domain containing 1-antisense 1 (DCST1-AS1) to enhance its transcript level. Furthermore, DCST1-AS1 can positively regulate *MYC* expression by sponging miR-873-5p. The positive feedback loop among DCST1-AS1, miR-873-5p, and *MYC* contributed to breast cancer cell proliferation [35]. Similarly, Linc00839 is another *MYC* activating lncRNA, and the ectopic expression of Linc00839 accelerated cell proliferation. Moreover, the inhibition of Linc00839 impeded tumor growth in nude mice [26]. Additionally, oncogenic lncRNA TROJAN has been identified to promote breast cancer progression. The downregulation of TROJAN suppressed breast cancer cell proliferation in vitro and tumor growth in vivo by regulating cyclin-dependent kinase 2 (CDK2) [15]. The enforced expression of ceramide synthase 6 antisense 1 (CERS6-AS1) facilitated the cell viability by enhancing the *CERS6* mRNA stability via interaction with the RNA-binding protein insulin-like growth factor binding protein-2 (IGFBP2) [9].

In the study reported by Hu et al., the lncRNA regulator of reprogramming (ROR) was expressed at a remarkable level in breast cancer tissues and cell lines [36]. Furthermore, the ROR was found to promote cell proliferation in vitro and tumor growth in mice, suggesting that it is an oncogenic lncRNA in breast cancer. Mechanistic investigations indicated that the ROR could enhance the tissue inhibitors of metalloproteinase 3 (*TIMP3*) transcription through demethylating the *TIMP3* promoter by binding to Mixed-lineage leukemia 1 (MLL1), thus accelerating breast cancer progression [36]. The interaction between Opa and protein 5-antisense RNA 1 (OIP5-AS1) significantly facilitates cell proliferation by repressing glyoxalase 1 (*GLO1*) [37]. In addition, HOXC-AS3 was also highly expressed in breast cancer tissues, and further researches point out that HOXC-AS3 affected the proliferation of breast cancer cells by targeting thymidine kinase 1 (*TK1*) [27].

Meanwhile, some lncRNAs have been reported to serve as the growth suppressors in breast cancer. For instance, Li et al. reported that the upregulation of GAS5 inhibited cell growth by reducing miR-196a-5p and repressing the PI3K/Akt pathway in breast cancer cells [20]. MT1JP was downregulated in breast cancer samples and its overexpression inhibited cell proliferation by downregulating miR-24-3p in breast cancer cells [39]. Heart and neural crest derivatives expressed by 2-antisense RNA 1 (HAND2-AS1) have been observed to reduce the cell growth of breast cancer cells by modulating miR-3118 and miR-1275 [40,41]. In addition, LINC01189 was reported to inhibit breast cancer cell growth and tumor formation in mice by sponging miR-586 and inactivating the Wnt/β-catenin pathway [42]. Yao et al. reported that MICAL2-1 was also downregulated in breast cancer tissues and its overexpression suppressed cell proliferation by targeting the miR-25/Dickkopf 3 (*DKK3*) axis [43]. It is likely that the diminished expression of insulin-like growth factor 2 antisense RNA (IGF2-AS) was observed in breast cancer specimens and cell lines. Moreover, ectopic IGF2-AS expression has been found to impair cell growth in breast cancer by repressing IGF2 and subsequently inhibiting the PI3K/AKT/mTOR signaling pathway [44]. LncRNA ZNFX1 antisense RNA 1 (ZFAS1) levels were downregulated in breast cancer tissues, and silencing ZFAS1 expression obviously promoted the cell proliferation of MDA-MB-231 by activating the signal transducer and activator of the transcription 3 (STAT3) pathway [45]. Additionally, LINC00641 was downregulated in breast cancer tissues compared with adjacent normal tissues. The overexpression of LINC00641 significantly blocked the cell proliferation in vitro and tumor growth in mice by absorbing miR-194-5p [46]. Collectively, these findings have certainly indicated the crucial roles of lncRNAs in regulating the cell proliferation of breast cancer. Therefore, blocking the uncontrolled growth of cancer cells by specifically targeting lncRNAs could provide an attractive treatment strategy for breast cancer.

## 4. LncRNAs Regulate Cell Apoptosis in Breast Cancer

Increasing evidence has highlighted the key roles of lncRNAs in regulating breast cancer cell apoptosis. For instance, LNC942 (LINC00942) could enhance the mRNA stability and expression level of chemokine receptor 4 (*CXCR4*) and cytochrome P450 1B1 (*CYP1B1*) by cooperating with N6-methyladenine (m6A)-related protein methyltransferase-like 14 (METTL14), which could eventually inhibit apoptosis in breast cancer cells [47]. The inhibition of DLX6-AS1 in MCF-7 breast cancer cells enhances the cell apoptosis process by directly sponging miR-505-3p to upregulate runt-related transcription factor 2 (*RUNX2*) [23]. Bao et al. reported that overexpressed CERS6-AS1 inhibited the breast cancer cell apoptosis by upregulating *CERS6* [9]. The silencing of LINC00673 in MDA-MB-231 and MDA-MB-453 breast cancer cells enhanced cell apoptosis by modulating apoptosis-related proteins [13]. The knockdown of LINC01121 has been identified to induce the apoptosis of breast cancer cells by repressing high-mobility group protein 2 (*HMGA2*) expressions [48]. Moreover, the inhibition of HOXC-AS3 in breast cancer cells was observed to induce cell apoptosis via the Y-box binding protein 1 (YBX1)/*TK1* axis [27]. SOX21-AS1 depletion has been found to promote cell apoptosis by inducing the proapoptotic proteins [32]. Likely, silencing ROR reduces Bcl-2 expression, but increases the expression of Bax and Cleaved caspase-3, indicating that lncRNA ROR may be involved in the apoptotic process in breast cancer cells [36].

On the other hand, other lncRNAs could promote cancer cell apoptosis. IGF2-AS upregulation has been demonstrated to promote apoptotic death in T47D breast cancer cells [44]. LncRNA FGF14 antisense RNA 2 (FGF14-AS2) is significantly reduced in breast cancer tissues, and its overexpression promotes apoptosis in breast cancer cells by regulating miR-205-5p [49]. Overexpressed PTENP1 has been reported to induce cell apoptosis through the miR-20a/*PTEN*/PI3K/AKT axis [19]. Thus, these studies emphasize the significant role of lncRNAs in regulating cell-related death in breast cancer.

## 5. LncRNAs Regulate Cell Migration, Invasion, and Metastasis

Through invasion, migration, and metastasis, tumor cells can move from lymphatic to circulatory systems and even distant organs, enhancing the malignancy of tumor cells. LncRNAs have been revealed to regulate the cell invasion and metastasis process in breast cancer. LINC01121 enhanced cell invasion and migration by sponging miR-150-5p to regulate the expression of *HMGA2* [48]. The inhibition of HOTAIR impaired cell migration and invasion by interacting with miR-601 in breast cancer [30]. In the study reported by Li et al., the invasive ability of breast cancer cells was significantly abolished after MIR210HG knockdown [24]. LNC942-overexpressed MCF-7 cells and SKBR3 cells exhibited enhanced migratory capability compared with the control groups [47]. Moreover, upregulated LINC00511 promoted cell invasion via the miR-185-3p/*E2F1* axis [14]. The inhibition of DLX6-AS1 attenuated the breast cancer cell invasion by repressing *RUNX2* [23]. HULC knockdown attenuated cell invasion and migration by regulating the miR-6754-5p/Ly6/PLAUR domain-containing protein 1 (*LYPD1*) axis in breast cancer cells [8]. Another lncRNA, differentiation antagonizing non-protein coding RNA (DANCR), which can be activated by tuftelin 1 (TUFT1), was upregulated in breast cancer tissues. Moreover, its inhibition suppressed the cell invasion of MDA-MB-231 by downregulating SRY-Box 2 (*SOX2*) [50].

Furthermore, Zhou et al. have revealed that the overexpression of RACGAP1P promoted cell migration, invasion, and metastasis in breast cancer. Mechanistically, RACGAP1P facilitated the migratory and invasive ability of breast cancer cells by targeting miR-345-5p [22]. The high expression of LINC00922 has been reported to facilitate the cell migration and invasion in vitro, as well as promote cancer cell metastasis in vivo. Mechanistically, LINC00922 recruited DNA methylases to the promoter of naked cuticle homolog 2 (*NKD2*), which induced the aberrant methylation of *NKD2*, helping to enhance the migratory and invasive ability of breast cancer cells [51]. In addition, the inhibition of LCPAT1 impaired the cell invasion and migration of MCF-7 and MDA-MB-231 breast cancer cells by decreasing *MFAP2* expression [33]. The knockdown of SOX21-AS1 also restrained the migration and invasion of breast cancer cells by modulating the miR-520a-5p/*ORMDL3* axis [32]. Dong et al. demonstrated that LOXL1-AS1 enhanced the migration, invasion, and lung metastasis of breast cancer cells by downregulating miR-708-5p [52].

In contrast, overexpressed lncRNA MICAL2-1 was found to impair the migration and invasion of breast cancer cells by modulating the miR-25/*DKK3* axis [43]. Overexpressed LINC01189 blocked the migration, invasion, and metastasis of breast cancer cells [42]. It is likely that LINC00641 has been found to inhibit cell migration and invasion by repressing miR-194-5p [46]. In addition, the upregulation of FGF14-AS2 impeded breast cancer cell migration and invasion [49]. Taken together, these reports demonstrated that lncRNAs are closely involved in breast cancer metastasis through various molecular mechanisms; a better understanding of breast-cancer-related lncRNAs will certainly improve our awareness of the initiation and development of cancer. Therefore, more researches on the potential use of specific lncRNAs as biomarker and therapeutic targets will shed new light on the clinical treatment of breast cancer.

## 6. LncRNAs Regulate the EMT Process in Breast Cancer

The EMT is a complicated cellular process whereby epithelial-like cells are transformed into mesenchymal cells, and the process is closely related to tumor metastasis. A variety of lncRNAs have been found to participate in EMT progression in breast cancer through diverse mechanisms. For instance, the inhibition of DLX6-AS1 was reported to increase the protein level of E-cadherin and decrease vimentin expression, thus repressing the EMT process in breast cancer cells [23]. Silencing lncRNA ZEB2-AS1 was found to impede cell invasion and migration in vitro and tumor lung metastasis in mice by targeting the EMT-related protein ZEB2 in breast cancer [25]. The ectopic expression of LINC00922 has been found to induce the EMT process in breast cancer cells by targeting *NKD2* [51]. Moreover, Fang et al. reported that TTN-AS1 could promote the EMT process in breast cancer cells by modulating the miR-139-5p/*ZEB1* axis [29]. The knockdown of DANCR reduced N-cadherin and vimentin. Nevertheless, DANCR displayed a sponge of miR-874-3p to derepress the expression level of *SOX2*, which enhanced the EMT in breast cancer cells [50]. Upregulated ROR has been found to promote EMT progression in breast cancer cells by elevating vimentin, N-cadherin, MMP2, and MMP9 [36]. Otherwise, SOX21-AS1 silencing in MCF7 and BT-20 cells repressed vimentin and N-cadherin expression, while its overexpression displayed the opposite effect, which indicates its promoting role in EMT progression in breast cancer [31]. Additionally, the silencing of OIP5-AS1 was observed to inactivate the EMT process in breast cancer cells through the miR-340-5p/*ZEB2* axis [38].

On the other hand, several lncRNAs can also inactivate the EMT process in breast cancer. The inhibition of ZFAS1 enhances the EMT process by activating the STAT3 pathway in MDA-MB-231 cells [45]. Guo et al. reported that LINC00261 exhibited a lower expression level in breast cancer tissues compared to non-tumor samples [53]. Functional experiments showed that the enforced expression of LINC00261 suppressed the EMT development in breast cancer cells by enhancing NME/NM23 nucleoside diphosphate kinase 1 (*NME1*) mRNA stability [53]. Additionally, the downregulation of NEAT1 induced the expression of N-cadherin and vimentin, but repressed that of E-cadherin, indicating that NEAT1 may inhibit the EMT process in breast cancer cells [34]. Collectively, these findings have highlighted the critical roles of lncRNAs in regulating the breast cancer EMT process. Although the roles of lncRNAs in modulating the EMT have been widely explored, there are still a larger number of lncRNAs that have not been fully elucidated, and some lncRNAs may act in opposite directions. Further studies are needed to gain a better understanding of the roles of lncRNAs as EMT regulators in breast cancer.

## 7. LncRNAs in Drug Resistance

Drug resistance is one of the most significant obstacles in the clinical treatment of breast cancer patients, accompanied by a lousy prognosis. Of note, various dysregulated lncRNAs have been shown to play central parts in breast cancer drug resistance. MT1JP overexpression specifically sensitized breast cancer cells to cisplatin treatment by targeting miR-24-3p [39]. LncRNA cyclin D1-interacting long noncoding RNA 1 (DILA1), which is overexpressed in tamoxifen-resistant breast cancer cells, can reduce the ubiquitination of cyclin D1, thus resulting in breast cancer cells being less sensitive to tamoxifen treatment [54]. As an oncogene, the cytoskeleton regulator RNA (CYTOR) can sponge miR-125a-5p to raise the level of serum response factor (*SRF*). Blocking CYTOR enhanced the sensitivity of breast cancer cells to tamoxifen stimulation [12]. In addition, lncRNA H19 was found to upregulate *Beclin-1* expression, thereby promoting autophagy and enhancing tamoxifen resistance in breast cancer cells [18].

Apart from the lncRNAs above, some other lncRNAs participate in breast cancer adriamycin (ADR) resistance. In MCF-7/ADR cells, the expression levels of lncRNA CBR3 antisense RNA 1 (CBR3-AS1) were increased compared with ADR-sensitive cells. Furthermore, CBR3-AS1 can repress miR-25-3p by acting as a ceRNA and subsequently upregulate the expression of *MEK4* and *JNK1*, thus promoting ADR resistance [55]. Likely, LOC645166 has been revealed to recruit NF-κB to increase the transcription level of GATA binding protein 3 (*GATA3*), thereby inducing the STAT3 signaling pathway, which eventually facilitated ADR resistance in breast cancer cells [56]. Linc00839 was significantly upregulated both in tamoxifen-resistant breast cancer cells and ADR-resistant breast cancer cells, and the inhibition of Linc00839 enhanced apoptosis induced by paclitaxel by targeting the PI3K/AKT pathway in breast cancer cells [26]. The knockdown of small nucleolar RNA host gene 7 (SNHG7) has been reported to increase the paclitaxel treatment efficiency in breast cancer cells by sponging miR-34a [57]. Additionally, Jin et al. reported that the inhibition of TROJAN reversed the resistance to the cyclin-dependent kinase 4/6 (CDK4/6) inhibitor in breast cancer cells by suppressing CDK2 [15].

In contrast, while overexpressed GAS5 can strengthen ADR sensitivity in vivo and in vitro, GAS5 acted as a sponge to miR-221-3p and subsequently drove the expression of dickkopf 2 (*DKK2*), which ultimately inactivated the Wnt/β-catenin pathway to weaken ADR resistance in breast cancer cells [21]. A lower histocompatibility leukocyte antigen complex P5 (HCP5) expression level was ascertained in cisplatin-resistant breast cancer cells, while overexpressed HCP5 weakened cisplatin resistance in vivo and in vitro by upregulating PTEN [58]. Above all, elucidating the regulatory mechanisms associated with lncRNAs in breast cancer chemotherapy resistance is essential to explore novel medicines for the treatment of chemo-resistant tumors. In addition, the lncRNA-based combined treatment scheme might be a promising strategy to overcome drug resistance in breast cancer treatment.

## 8. Roles of circRNAs in Breast Cancer

Accumulating a body of evidence indicates that circRNAs play vital roles in the development and process of breast cancer (Table 2). For instance, circACTN4 (hsa_circ_0050900) was highly expressed in breast cancer tumor, and its expression level negatively correlated with tumor stage and overall survival rate in breast cancer patients [64]. The silencing of circACTN4 inhibited cell proliferation, migration, and invasion. Mechanistic studies have showed that circACTN4 can disrupt the interaction between far upstream element binding protein 1 (FUBP1) and poly (U) binding splicing factor 60 (FIR) by competitively binding to FUBP1 and subsequently enhancing the *MYC* transcription level, which contributes to breast cancer progression [64]. Wu et al. illustrated that circ_0000511 was upregulated in breast cancer samples and cell lines [65]. The downregulation of circ_0000511 inhibited cell proliferation, migration, invasion, and enhanced cell apoptosis by regulating circ_0000511/miR-326/*TAZ* [65]. The circRNA zinc finger RNA binding protein (circ_ZFR) has been shown to competitively bind to miR-223-3p, thereby enhancing fatty acid binding protein 7 (*FABP7*) expression, and promoting the cell growth, migration, invasion, and EMT process in breast cancer [66]. Moreover, circMMP11 is highly expressed in breast cancer tissues compared to non-tumor tissues, and its downregulation in MCF-7 cells inhibited the cell proliferation, migration, and EMT process by inducing the tumor suppressor miR-625-5p and repressing *ZEB2* [67]. Recently, Yang et al. have ascertained a novel circRNA, circWSB1 (circ_0007716), which is highly expressed in breast cancer tissues, and can be activated by HIF-1α and impair the stability of the p53 protein by disturbing the interaction between p53 and ubiquitin-specific peptidase 10 (USP10) [68]. These factors eventually promoted cell proliferation and tumor growth in vitro and in vivo, which drove the progression of breast cancer [68]. Based on circRNA sequencing, circCDYL2 has been identified to be elevated in trastuzumab-resistant breast cancer tissues and cell lines. Further studies revealed that circCDYL2 enhanced trastuzumab resistance by activating the AKT and ERK pathways [69]. Liu et al. reported that circ_0006528 could contribute to paclitaxel resistance in breast cancer cells by regulating the miR-1299/cyclin-dependent kinase 8 (*CDK8*) axis [70].

circAHNAK1 was downregulated in breast cancer tissues; was negatively related to tumor size, tumor state, and lymph node metastasis; and was correlated with better outcomes in breast cancer patients. Additionally, enforced circAHNAK1 expression inhibited cell proliferation, migration, and invasion by targeting the miR-421/*RASA1* axis [71]. In another study, Guo et al. reported that circKDM4B (has_circ_0002926) was downregulated in breast cancer tissues and its overexpression inhibited cell migration and invasion in vitro and tumor growth and metastasis in vivo, indicating its tumor suppressor role in breast cancer [72]. Thus, the evidence above has highlighted the crucial regulatory roles of circRNAs in the process and pathogenesis of breast cancer, reflecting them as the potential diagnostic and therapeutic targets for breast cancer. Therefore, more studies are needed to acquire a clear understanding of the biological functions, molecular mechanisms, and clinical applications of circRNAs in breast cancer.

## 9. Conclusions

Over the last decade, numerous ncRNAs have been revealed to be involved in diverse physiological and pathophysiological processes, including breast cancer. As the essential subtype of ncRNAs, lncRNAs and circRNAs in breast cancer have garnered increasing attention. At the present review, we summarized the functions of lncRNAs and circRNAs and underlying mechanisms by which lncRNAs and circRNAs have impacts on breast cancer. These dysregulated lncRNAs and circRNAs are closely related to cell apoptosis, proliferation, migration, and drug resistance, resulting in the initiation and progression of breast cancer, reflecting them as the potential therapeutic targets for breast cancer treatment. Currently, lncRNA-based therapeutics such as antisense oligonucleotides (ASOs) and small interfering RNAs (RNAi) for breast cancer treatment have been widely investigated. Moreover, the relationship between lncRNAs and the clinical pathological features of breast cancer patients indicate lncRNAs as potential biomarkers for the diagnosis and prognosis of this disease.

Along with the dysregulated key genes to contribute to tumor initiation and progression, cancer cells also become supplemented from the surrounding environment, which is are called as the tumor microenvironment (TME), to promote cell growth and evade immune suppression. The combination of cancer cells and TME can be regard as a whole eco-system, which eventually drives the initiation and progression of multiple cancers [73,74]. The TME is a complex system comprising multiple types of cells and non-cellular constituents [75]. Recently, a number of studies have highlighted the critical roles of TME in breast cancer. Of note, lncRNAs also play regulatory roles in breast cancer by interacting with the TME. LncRNAs can interact with tumor-associated macrophages (TAMs) in the TME of breast cancer [76]. LncRNA linc00514 is elevated in breast cancer tissues and its overexpression enhances the M2 polarization of TAMs by activating the Notch pathway, which contributes to breast cancer metastasis [77]. Small nucleolar RNA host gene 1 (SNHG1) can also manipulate the immune-related cells in the TME. Pei et al. demonstrated that SNHG1 suppressed the differentiation of Treg cells by sponging miR-448 to upregulate the expression of indoleamine 2,3-dioxygenase (*IDO*), enhancing the mediated immune escape of breast cancer cells [78]. Above all, the interplay between lncRNAs and TME may provide a novel understanding of the breast cancer progression, contributing to developing strategies for the clinical treatment of breast cancer patients.

However, there are still some questions that should be asked about the lncRNA-based diagnoses and therapies for breast cancer patients. Firstly, lncRNAs associated with breast cancer cannot work on their own to obtain accurate diagnostics. The diagnostic strategies that combine lncRNAs and other biomarkers may be more achievable and may facilitate early diagnosis for breast cancer. Secondly, studies have shown that lncRNAs can regulate multiple cellular processes through diverse mechanisms. Thus, detailed molecular mechanisms by which lncRNAs function should be fully uncovered individually. Thirdly, each lncRNA may have multiple target genes, and off-target effects may cause serious negative impacts on lncRNA-based therapeutics. In addition, efficient delivery methods are crucial to the satisfactory clinical treatment of breast cancer patients. Recently, the application of novel materials and techniques for advanced therapeutical delivery systems may improve this situation. Lastly, researches about lncRNAs and circRNAs, and their ability to module breast cancer progression by targeting the TME, lack any depth; thus, further studies should pay more attention to this aspect.

In conclusion, with various novel techniques, such as high-throughput sequencing, applied into lncRNA investigations, the functions and modulating mechanisms of lncRNAs in breast cancer may be fully discovered in the future. Moreover, lncRNA-based therapies could eventually bring a better prognosis to patients with breast cancer.

## Figures and Tables

**Table 1:** Regulatory roles and clinical features of representative lncRNAs in breast cancer.

LncRNAs	Expression	Related Clinicopathologic Features	Functions	Mechanisms	References
HULC	up	Reduced overall survival and poor tumor stage	Promotes invasion and migration	miR-6754-5p/*LYPD1*	[8]
CERS6-AS1	up	Advanced tumor grade and clinical stage, and poor prognosis	Promotes proliferation and inhibits apoptosis	*CERS6*	[9]
H19	up	Lymphatic metastasis and poor prognosis	Enhances cell growth, metastasis, and drug resistance	miR-138/*SOX4*, *Beclin-1*	[11,18]
CYTOR	up	/	Promotes drug resistance	miR-125a-5p/*SRF*	[12]
LINC00673	up	Tumor size and poor survival	Promotes proliferation and inhibits apoptosis	miR-515-5p/MARK4, Hippo	[13]
LINC00511	up	Larger tumor size, advanced TNM stage, and lymph node metastasis	Promotes invasion	miR-185-3p/*E2F1*	[14]
TROJAN	up	Larger tumor size, worse pathologic grade, and postoperative recurrence	Increases proliferation and drug resistance	NKRF, CDK2	[15]
PTENP1	down	Late tumor stage	Inhibits proliferation and induces apoptosis	miR-20a/*PTEN*/PI3K/AKT	[19]
GAS5	down	Lager tumor size, worse histological grade, and lymph node metastasis	Inhibits cell growth and drug resistance	miR-196a-5p, miR-221-3p/*DKK2*	[20,21]
RACGAP1P	up	Tumor stage, distant metastasis, and lymph node metastasis	Enhances migration and invasion	miR-345-5p/*RACGAP1*	[22]
DLX6-AS1	up	Tumor size, lymph node metastasis, and clinical stage	Promotes invasion, migration, and the EMT; inhibits apoptosis	miR-505-3p/*RUNX2*	[23]
MIR210HG	up	Lymph node metastasis and distance metastasis, and poor overall survival	Promotes cell proliferation and invasion	miR-1226-3p/*MUC1-C*	[24]
ZEB2-AS1	up	Lymph node and distant metastasis, and reduced overall survival	Promotes migration and invasion	ZEB2	[25]
LINC00839	up	Advanced TNM stage and lymph node metastasis	Promotes cell growth and drug resistance; inhibits apoptosis	PI3K/AKT	[26]
HOXC-AS3	up	Reduced survival time, HR = 6.742 (*p* < 0.01), lymph node metastasis, and advanced TNM stage	Promotes cell growth; inhibits apoptosis	YBX1/*TK1*	[27]
TTN-AS1	up	Lymph node invasion and metastasis, and advanced TNM stage	Promotes proliferation	miR-524-5p/*RRM2*, miR-139-5p/*ZEB1*	[28,29]
HOTAIR	up	Advanced TNM stage, lymph node metastasis, and worse survival rate	Increases proliferation, migration, and invasion	miR-601/*ZEB1*	[30]
SOX21-AS1	up	Larger tumor size, advanced TNM stage, poor differentiation grade, lymph node metastasis, reduced survival, AUC = 0.93 (*p* < 0.05), and can be used for the diagnosis and prognosis of BC	Enhances cell proliferation, migration, and the EMT; inhibits apoptosis	PI3K/AKT, miR-520a/5p/*ORMDL3*	[31,32]
LCPAT1	up	High-grade differentiation, advanced TNM stage, and poor overall survival	Promotes proliferation, migration, and invasion	*MFAP2*	[33]
NEAT1	up	Poor overall survival	Enhances proliferation and the EMT	miR-410-3p/*CCND1*	[34]
DCST1-AS1	up	Lymph node metastasis and poor tumor grade	Promotes cell growth	miR-873-5p/*MYC*	[35]
ROR	up	/	Promotes cell growth and the EMT; inhibits apoptosis	TIMP3	[36]
OIP5-AS1	up	Reduced overall survival	Promotes proliferation and the EMT	miR-216a-5p/*GLO1*, miR-340-5p/*ZEB2*	[37,38]
MT1JP	down	Advanced TNM stage and poor overall survival	Inhibits proliferation and drug resistance	miR-24-3p	[39]
HAND2-AS1	down	Increased overall survival and lower postoperative recurrence	Inhibits proliferation	miR-3118/*PHLPP2*, miR-1275/*SOX7*	[40,41]
LINC01189	up	/	Inhibits proliferation and migration	miR-586/*ZEB1*, Wnt/β-catenin	[42]
MICAL2-1	/	/	Inhibits cell growth, migration, and invasion	miR-25/*DKK3*	[43]
IGF2-AS	down	Smaller tumor size, increased overall survival, AUC = 0.8003 (*p* = 0.0002), a promising biomarker for the diagnosis and prognosis of breast cancer	Impairs cell growth and induces cell apoptotic death	IGF2, PI3K/AKT/mTOR	[44]
ZFAS1	down	/	Inhibits proliferation and the EMT	STAT3	[45]
LINC00641	down	Smaller tumor size, less lymph node metastasis, and better clinical stage	Inhibits proliferation, migration, and invasion	miR-194-5p	[46]
LNC942	up	Poor tumor grade and larger tumor size	Promotes migration and inhibits apoptosis	*CXCR4,CYP1B1*	[47]
LINC01121	/	/	Increases invasion and migration; inhibits apoptosis	miR-150-5p/*HMGA2*	[48]
FGF14-AS2	down	Smaller tumor size and increased overall survival	Inhibits migration and invasion; induces apoptosis	miR-205-5p	[49]
DANCR	up	Reduced overall survival	Promotes proliferation, migration, and the EMT	miR-874-3p/*SOX2*	[50]
LINC00922	up	Poor overall survival	Increases invasion and migration; promotes the EMT	*NKD2*	[51]
LOXL1-AS1	up	Advanced TNM stage and lymph node metastasis	Increases proliferation, migration, and invasion; inhibits apoptosis	miR-708-5p	[52]
LINC00261	down	Advanced clinical stage and lymph node metastasis	Inhibits the EMT	*NME1*	[53]
DILA1	/	/	Enhances drug resistance	Cyclin D1	[54]
CBR3-AS1	up	Larger tumor size, advanced pathological stage, and poor overall survival	Promotes drug resistance	miR-25-3p/*MEK4*/*JNK1*	[55]
LOC645166	/	/	Promotes drug resistance	*GATA3*	[56]
SNHG7	up	Larger tumor size, advanced tumor stage, and poor overall survival	Promotes drug resistance	miR-34a	[57]
HCP5	/	/	Increases drug resistance	PTEN	[58]

Abbreviations: HR: hazard ratio; AUC: area under the curve.

**Table 2:** Regulatory roles of representative circRNAs in breast cancer.

LncRNAs	Samples	Expression	Functions	Mechanisms	Reference
circACTN4	80 paired BCT and BCN tissues, MCF-7 and SK-BR-3 cell lines	up	Enhances cell growth, migration, and invasion metastasis	FUBP1	[64]
circ_0000511	50 paired BCT and BCN tissues, MCF-7and MDA-MB-468 cell lines	up	Inhibits cell proliferation, migration, and invasion; enhances cell apoptosis	miR-326/*TAZ*	[65]
circ_ZFR	50 paired BCT and BCN tissues, MCF-7 and MDA-MB-231 cell lines	up	Promotes growth, migration, invasion, and the EMT	miR-223-3p/*FABP7*	[66]
circMMP11	34 paired BCT and BCN tissues, MCF-7 and MDA-MB-231 cell lines	up	Promotes proliferation, migration, invasion, and the EMT	miR-625-5p/*ZEB2*	[67]
circWSB1	100 paired BCT and BCN tissues, MDA-MB-453 and MCF-7 cell lines	up	Promotes proliferation	p53	[68]
circAHNAK1	Unpaired BCT and BCN tissues, MDA-MB-231 and BT-549 cell lines	down	Inhibits proliferation, migration, and invasion	miR-421/*RASA1*	[71]
circKDM4B	Unpaired BCT and BCN tissues, MDA-MB-231 and MDA-MB-468 cell lines	down	Inhibits migration and invasion	miR-675/*NEDD4L*	[72]
circCDYL2	Unpaired trastuzumab-sensitive and -resistant BCT tissues, BT-474 and SK-BR-3 cell lines	up	Enhances drug resistance	AKT,ERK	[69]
circ_0006528	48 paired BCT and BCN tissues, BT-549 and ZR-75-30 cell lines	up	Enhances drug resistance	miR-1299/*CDK8*	[70]

BCT: breast cancer tissues; BCN: breast cancer paired and adjusted for normal tissues.
